# In Vivo Evaluation of Innovative Gadolinium-Based Contrast Agents Designed for Bioimaging Applications

**DOI:** 10.3390/polym16081064

**Published:** 2024-04-11

**Authors:** Sorina Nicoleta Voicu, Cecilia Virginia Gheran, Cornel Balta, Anca Hermenean, Maité Callewaert, Françoise Chuburu, Anca Dinischiotu

**Affiliations:** 1 Department of Biochemistry and Molecular Biology, Faculty of Biology, University of Bucharest, 050095 Bucharest, Romania; sorina.voicu@bio.unibuc.ro (S.N.V.); virginia.gheran@gmail.com (C.V.G.); 2 Department of Experimental and Applied Biology, Institute of Life Sciences, Vasile Goldis Western University of Arad, 86 Rebreanu, 310414 Arad, Romania; balta.cornel@uvvg.ro (C.B.); hermenean.anca@uvvg.ro (A.H.); 3 Institut de Chimie Moléculaire de Reims, CNRS UMR 7312, Université de Reims Champagne-Ardenne URCA, CEDEX 2, F-51685 Reims, France; maite.callewaert@univ-reims.fr (M.C.); francoise.chuburu@univ-reims.fr (F.C.)

**Keywords:** gadolinium-based contrast agents, nanohydrogels, magnetic resonance imaging

## Abstract

The aim of this study was the investigation of biochemical and histological changes induced in different tissues, as a result of the subcutaneous administration of Gd nanohydrogels (GdDOTA⸦CS-TPP/HA) in a CD-1 mouse strain. The nanohydrogels were obtained by encapsulating contrast agents (GdDOTA) in a biocompatible polymer matrix composed of chitosan (CS) and hyaluronic acid (HA) through the ionic gelation process. The effects of Gd nanohydrogels on the redox status were evaluated by measuring specific activities of the antioxidant enzymes catalase (CAT), glutathione peroxidase (GPx), and superoxide dismutase (SOD), as well as oxidative stress markers, such as reduced glutathione (GSH), malondialdehyde (MDA), advanced oxidation protein products (AOPP), and protein-reactive carbonyl groups (PRCG), in the liver, kidney, and heart tissues. The nitrosylated proteins expression were analyzed with Western Blot and the serum biochemical markers were measured with spectrophotometric methods. Also, a histological analysis of CD-1 mouse tissues was investigated. These results indicated that Gd nanohydrogels could potentially be an alternative to current MRI contrast agents thanks to their low toxicity in vivo.

## 1. Introduction

Magnetic resonance imaging (MRI) is an important diagnostic method with high spatial resolution that can be used for soft tissue analyses due to its capacity to provide a very good contrast without the need for ionizing radiation [1]. 

Gadolinium (Gd)-based contrast agents (GBCAs) are the most widespread in the contrast-enhanced magnetic resonance imaging market, due to the optimal magnetic properties of Gd^3+^, i.e., a high magnetic moment associated with the presence of seven unpaired electrons and long electronic relaxation times [2]. Being paramagnetic, it interacts magnetically with water molecules in its vicinity, inducing signal changes in the magnetic resonance images and generating a contrast in them [3]. Gd^3+^ complexes present high thermodynamic stability, high aqueous solubility, stability of oxidation state, and low osmolality [4].

The use of GBCAs is focused on the detection and characterization of focal visceral organ lesions, cancer staging, and the evaluation of infectious and inflammatory processes in different organ systems [5].

Due to its size similarity with Ca^2+^, Gd^3+^ can inhibit voltage-gated Ca^2+^ channels and the activities of calcium-dependent enzymes [6].

As Gd^3+^ is toxic, it can be used only in the presence of a chelating substance, with linear or cyclic morphology, that binds strongly to this heavy metal ion to improve the stability, solubility, and safety of GBCA [7]. Thus, GBCAs can be divided into linear or macrocyclic compounds based on the molecular structure of the organic ligand and in nonionic and ionic groups depending on their net charge in aqueous solutions [8]. The stability of GBCAs depends on their kinetic and thermodynamic stability, with macrocyclic chelates being much more stable than linear (acyclic) ones [9]. Their high selectivity, stability, and structural preorganization render them valuable for a wide range of applications, from metal ion extraction and sensing to biomedical imaging and therapy [9].

They are mainly excreted via the kidneys and the liver [10] and can accumulate in the tissues of patients who were exposed several times to GBCAs [11].

In this context, to increase the safety of these diagnostic tools, the development of systems with higher relaxivity could decrease the doses administered for clinical purposes (generally 0.1 mmol of Gd per kilogram of body weight). Several strategies have been tested in the last years towards this goal. They are based on the Solomon–Bloembergen–Morgan theory, which states that controlling the rotational correlation time of contrast agents, modulated by increasing object mass, and the exchange rate of water molecules around the gadolinium center has a positive impact on increasing contrast agent relaxivity. Biodegradable macromolecular protein polymers obtained via amide bond formation between lysine-containing random-coil protein and Gd(III)DO3A chelates are a first example. The resulting bioconjugates exhibit greater relaxivity per gadolinium center than chelates alone (relaxivity up to 14 mM^−1^ s^−1^ at 60 MHz, which is about 3–5 times higher than that for clinically used small-molecule contrast agents). The random coil secondary structure of the protein may adopt a globular formation where the different sections can come into contact with each other, resulting in steric hindrance of the Gd(III) chelators and increased relaxivity [12]. Another example corresponds to the formation of columnar aggregates obtained via polymerization in water of Gd(III)DTPA grafted C3-symmetric discotic amphiphiles. These supramolecular assemblies have a relaxivity per Gd(III) center of 12 mM^−1^ s^−1^ at 60 MHz, a value that is, again, well above those of isolated chelates (3.9 mM^−1^ s^−1^ at 60 MHz for Gd(III)DTPA) [13]. Molecular self-organization into fibrillar structures of polyaromatic peptides conjugated to Gd(III) chelates has more recently been described for its ability to form hydrogels in which the relaxivity of Gd chelates is also exalted [14,15,16]. Relaxivities of corresponding DOTA(Gd)-PEG8-(FY)3 and DTPA(Gd)-PEG8-(FY)3 self-assembled contrast agents are similar and close to 12 mM^−1^ s^−1^ (at 20 MHz), and these high relaxivity values can be attributed to pH-induced aggregation of the system in hydrogel form [14]. Hydrogels can also be obtained as nanogels [17] via supramolecular assemblies between biopolymers of opposite charges such as chitosan and hyaluronic acid. Chitosan is derived from chitin, a naturally occurring biopolymer found in the exoskeletons of crustaceans, and is a linear, positively charged polysaccharide [18]. Hyaluronic acid is a natural component of the extracellular matrix and is found in high concentrations in connective tissues, making it highly biocompatible and biodegradable [19,20]. Hyaluronic acid is a linear, negatively charged polymer [21]. Gd(III) chelates embedded in chitosan/hyaluronate-based nanogels presented a high relaxivity increase (relaxivity from 20 to 60 mM^−1^ s^−1^ at 20 MHz according to Gd(III) chelate) compared to nonencapsulated chelates (6.5 to 9 times higher) [2,22].

Considering this scientific context, in this paper we have investigated the potential toxicity of gadolinium nanohydrogels (GdDOTA⊂CS-TPP/HA) injected in mice at the level of the liver, kidney, and heart, analyzing the biochemical and histological alterations.

## 2. Materials and Methods

### 2.1. Synthesis and Characterization of GdDOTA⊂CS-TPP/HA Nanogels

The chitosan (CS) used in this study was purchased from Sigma-Aldrich (Saint Louis, MO, USA) and sourced from shrimp shells (Mn: 51,289 Da; Mw: 88,148 Da; PdI (= Mw/Mn): 1.72; viscosity = 33 mPa·s in 1% acetic acid, 20 °C). A deacetylation degree (DD) of 86% was determined with ^1^H NMR spectroscopy.

The stock solution of chitosan was obtained by solubilizing 2.5 mg·mL^−1^ in a 10% (*m*/*v*) citric acid aqueous solution and stirring overnight. Nanosuspensions were then freeze-dried, using glucose 15% (*m*/*v*) as a cryoprotectant. Nanoparticle average hydrodynamic diameters (DH) and polydispersity indexes (PdI) were determined with dynamic light scattering (DLS) (Malvern Zetasizer Nano-ZS, Malvern Instruments, Worcestershire, UK). A more detailed procedure was described in our previous works [19,20].

### 2.2. Experimental Models In Vivo CD-1 Albino Mice

The animal studies received ethical approval from the Ethical Committee of Vasile Goldis Western University of Arad (Approval no. 14, 27 October 2015). All experimental procedures were in strict compliance with the Declaration of Helsinki and were carried out in accordance with European and national standards for laboratory animal research. In this study, adult male CD1 mice weighing 30–35 g were used with a consistent nutritional plan based on typical rodent dietary requirements and were subjected to a stable 12 h cycle of light and darkness. The habitat’s conditions were precisely controlled, with the temperature maintained near 23 °C and relative humidity around 50%. Throughout the duration of this study, the mice were allowed unrestricted access to both food and water.

Animals were divided into 2 groups (6 per group): a control group injected subcutaneously in the center of the footpad with MilliQ ultrapure water and a group of mice injected subcutaneously with 0.07 μmoles Gd/30 µL of gadolinium nanohydrogels (GdDOTA⊂CS-TPP/HA). At 24 h and 48 h after injection, respectively, the mice were euthanized, and the liver, kidney, and heart were harvested and preserved at −80 °C until biochemical tests were performed.

**Figure 1:**
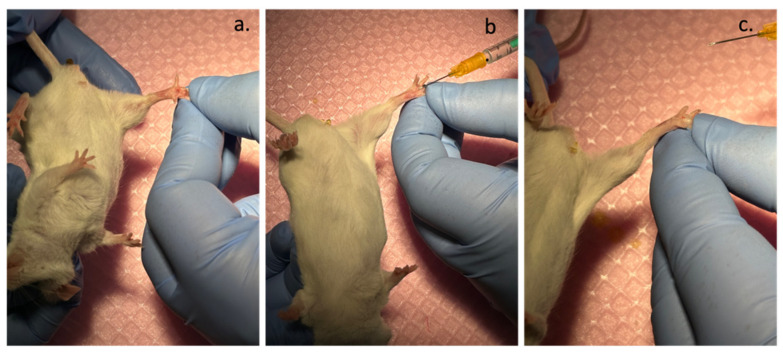
Experimental design. (**a**) Before administration; (**b**) injection; and (**c**) injection site after administration.

The experimental design can be found in Figure 1.

### 2.3. Biochemical Analyses

#### 2.3.1. Serum Biochemical Analyses

After 24 and 48 h post administration of nanogels, the blood samples were collected and centrifuged at 3500 rpm for 10 min. Then, the following markers were analyzed: aspartate transaminase (TGO/AST), alanine aminotransferase (TGP/ALT), lactate dehydrogenase (LDH), alkaline phosphatase (ALP), gamma-glutamyltranspeptidase (GGT), creatinine (CREA), and creatine kinase-MB (CK-MB), using a Mindray BS-120 Chemistry Analyzer (Shenzen Mindray Bio-Medical Electronics Co., Ltd., Nanshan, Shenzhen, China), using Chema Kits (Chema Diagnostica, Monsano, Italy).

#### 2.3.2. Preparation of the Total Tissue Extract

The tissues were thawed and homogenized on ice in Tris-EDTA buffer solution 0.1 M, pH 7.4, at an extraction ratio of 1 g:10 mL with the sonicator type Hielscher Ultrasonic processor UP50H (Hielscher Ultrasound Technology, Teltow, Germany). Then, supernatants were centrifuged at 10,000× *g* for 30 min at 4 °C. The total tissue extracts thus obtained were aliquoted for use in the subsequent biochemical analyses. The protein concentration of the tissue extracts was determined with the Lowry method, using bovine serum albumin (BSA) as a standard [23].

#### 2.3.3. Oxidative Stress Markers

##### Malondialdehyde (MDA) Assay

The level of malondialdehyde (MDA) in samples was determined according to the method described by Del Rio et al. [24], using thiobarbituric acid (TBA).

After each interval of treatment, the samples were analyzed. A volume of 200 μL of appropriately diluted tissue lysate was homogenized with 700 μL of 0.1 M HCl followed by incubation for 20 min at room temperature. Then, 900 µL of 0.025 M thiobarbituric acid (TBA) were added, and the mixture was incubated at 37 °C for 65 min. Finally, 400 µL of lysis buffer were pipetted into the samples to be analyzed, and in the samples used for the calibration curve, a volume of 400 µL of bovine serum albumin (BSA) was added. Fluorescence of MDA-TBA adducts was quantified (λex/em = 520/549 nm) using a Jasco FP-750 fluorimeter (Tokyo, Japan) and Spectra Manager software (version 1.10.00). The concentration of MDA was calculated using a calibration curve with 1,1,3,3-tetramethoxypropane in the range 0.5–5 μM, and the results were expressed in nmoles MDA/mg protein.

##### Reduced Glutathione (GSH) Assay

The concentration of reduced glutathione (GSH) was analyzed using the Glutathione Assay Kit (Sigma Aldrich, Saint Louis, MO, USA).

The tissue lysate obtained after treatment with GdDOTA nanohydrogels was deproteinized with 5% 5-sulfosalicylic acid (SSA) and subjected to centrifugation at 10,000 rpm at 4 °C for 10 min. Then, a volume of 10 µL of supernatant was transferred to a 96-well plate and incubated with 150 µL of reaction mixture (8 mL of 100 mM potassium phosphate buffer, pH 7.0, 1 mM EDTA–Assay Buffer) and 228 µL of 5,5-dithio-bis-(2-nitrobenzoic acid (DTNB) 1.5 mg/mL, at room temperature, for 5 min. The optical density of 2-nitro-5-thiobenzoic acid (TNB) was measured spectrophotometrically at 412 nm with the TECAN GENios Multireader (Tecan Trading AG, Männedorf, Switzerland). The concentration of GSH was calculated using a calibration curve made from a 10 mM GSH stock solution, and the results were expressed in nmoles GSH/mg protein.

##### Assessment of Advanced Protein Oxidation Products (AOPP)

The level of advanced protein oxidation products (AOPP) was assessed according to the method of Witko-Sarsat et al. [25] using a standard curve obtained with chloramine T up to 100 μM [26]. A volume of 200 µL of tissue lysate (diluted appropriately) and 10 µL of 1.16 M potassium iodide were homogenized for 5 min at room temperature. Then, a volume of 20 µL of glacial acetic acid was added and the mixture was vortexed again for 30 s. The absorbance was then immediately measured at 340 nm using the TECAN GENios Multireader, and the results were expressed in nmoles AOPP/mg protein.

##### Carbonyl Groups (PCG) Concentration

The protein carbonyl groups (PCG) concentration was determined according to the Field method [27] by measuring the hydrazones resulting from the reaction between 2,4-dinitrophenylhydrazine (DNPH) and protein carbonyls. The tissue lysates were incubated with 10 mM DNPH for one hour at room temperature in the dark. After protein precipitation with ice-cold 20% trichloroacetic acid (TCA), the samples were centrifuged, and the pellet was washed two times with an ethanol–ethyl acetate mixture. The absorbance of the carbonyl–DNPH products were measured at 370 nm and the concentration of PCG was calculated using the molar extinction coefficient, ε_DNPH_ = 22,000 M^−1^ cm^−1^. The concentration of PCG was related to the total protein concentration of the sample, and it was expressed in nmoles/ mg protein.

#### 2.3.4. Analyses of the Activities of Antioxidant Enzymes

##### Superoxide Dismutase (SOD) Activity Assay

The superoxide dismutase (SOD) activity was measured according to the method described by Paoletti et al. [28], based on the enzyme’s ability to catalyze the transformation reaction of superoxide anions into molecular oxygen and hydrogen peroxide. The method consisted of a sequence of chemical reactions, which generate superoxide anions (O_2_) from molecular oxygen by oxidizing β-mercaptoethanol in the presence of ethylenediaminetetraacetic acid (EDTA) and MnCl_2_. The reaction speed is determined by the decrease in optical density at 340 nm, as a result of NADH oxidation. The SOD activity in the samples is directly proportional to the inhibition rate of NADH oxidation. One unit of enzyme activity represents the amount of enzyme required to inhibit the oxidation rate of NADH by 50%. The optical density was read spectrophotometrically at 340 nm using the TECAN GENios Multireader.

##### Catalase (CAT) Activity Assay

Catalase (CAT) activity was measured according to the spectrophotometric method described by Aebi [29]. One enzyme unit decomposes one µmole of H_2_O_2_ in one minute at 25 °C and a pH of 7.0.

The absorbance was read at 240 nm using the Jasco spectrophotometer (Jasco, Tokyo, Japan), for 1 min after the addition of H_2_O_2_, with the reading interval being 25 s. The variation of the optical density 240 nm/min was calculated from the portion of the curve where the speed increases proportionally over time.

##### Glutathione Peroxidase (GPX) Activity Assay

The glutathione peroxidase (GPx) activity was measured according to the method described by Beutler et al. [30], based on the ability of GPx to catalyze the reduction reaction of peroxides in the presence of GSH.

The principle of the method is based on monitoring the formation of GSSG, which is continuously reduced by adding an excess of glutathione reductase, generating a constant level of GSH. The quantitative determination of the formed GSSG is realized indirectly, by measuring the concomitant oxidation of NADPH at 340 nm, in the reaction catalyzed by glutathione reductase.

The optical density was measured using the Perkin Elmer Lambda 25 spectrophotometer (Perkin, Norwalk, CT, USA) and the specific glutathione peroxidase activity was expressed in U/mg protein. One unit of GPx was defined as the amount of enzyme required to consume one µmole NADPH in one minute at 25 °C.

### 2.4. Western Blot Analyses

#### Western Blot Analysis of Anti-Nitrotyrosine Proteins

To highlight the protein expression modifications of the nitrosylated proteins, the Western Blot technique was used.

A quantity of 60 µg of protein was loaded into each well. A volume of 10 µL of See Blue Plus2 Prestained Standard 1× was used as a molecular mass marker. Protein migration was carried out at 90 V constant, for 2 h at 25 °C in Tris 0.05 M, glycine 0.05 M, and SDS 0.1% migration buffer.

After migration in the polyacrylamide gel, the proteins were transferred to the PVDF membrane (activated in 100% methanol) in the presence of a transfer buffer (Tris 25 mM, glycine 192 mM, and methanol 20%) at 350 mA, for 3 h 45 min, and at 4 °C.

After transfer, the membranes were washed 2 times with 20 mL of distilled water and then blocked in 10 mL of blocking buffer. The membranes were developed using monoclonal primary antibody mouse anti-nitrotyrosine obtained from Invitrogen (Carlsbad, CA, USA), respectively, for the determination of nitrosylated proteins. The Western Breeze Chromogenic Immunodetection (Invitrogen) kit with anti-mouse secondary antibodies coupled with alkaline phosphatase was used for the detections. The chromogenic substrate BCIP (5-bromo-4 chloro-3-indolyl-1 phosphate)/NBT (nitro blue tetrazolium) was maintained for 1 h until the appearance of the bands, which were later visualized and quantified with the ImageLab program from BioRad (version 6.1.0, Hercules, CA, USA).

### 2.5. Histological Analyses

The liver, heart, and kidney specimens were first fixed in 4% paraformaldehyde in a phosphate buffer solution (PBS) and subsequently embedded in paraffin to prepare them for histological examination. Following this, 5 μm thick sections were dyed using hematoxylin and eosin (H&E). After staining, the samples were examined under an Olympus BX43 microscope (Olympus, Tokyo, Japan), and an Olympus XC30 digital camera was used to capture high-resolution images of the chosen sections.

### 2.6. Statistical Analysis

The statistical analyses were performed by applying the Student’s *t*-test (TTEST function, Microsoft Excel, 2013). The results obtained were calculated as mean value ± SD (n = 6) and expressed relative to the corresponding control. The results are represented as the statistical significance: * *p* < 0.05 (significant, compared to control); ** *p* < 0.01 (distinctly significant, compared to control), *** *p* < 0.001 (very significant, compared to control); # *p* < 0.05 (significant, between the two time intervals); and ### *p* < 0.001 (highly significant, between the two time intervals).

## 3. Results

### 3.1. Physico–Chemical Characteristics of GdDOTA-Based Nanogels

GdDOTA nanohydrogels (NGs) were obtained using an ionotropic gelation process according to our previous works (Table 1) [19,20,31].

### 3.2. In Vivo Studies on CD-1 Albino Mice

#### Evaluation of Histological Changes Induced with Gadolinium Nanohydrogels (GdDOTA⸦CS-TPP/HA) in the Liver, Heart, and Kidney Tissues

**Figure 2:**
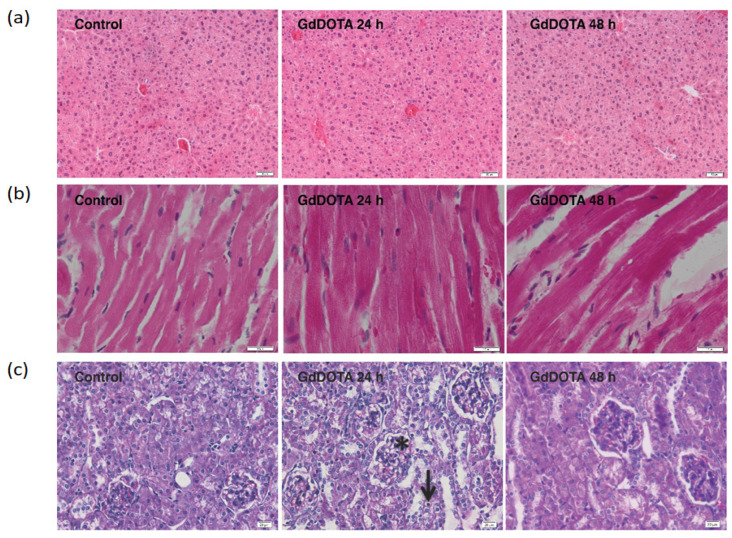
The histological aspect of the (**a**) liver, (**b**) myocardial tissue, and (**c**) kidney following administration of GdDOTA⊂CS-TPP/HA for 24 and 48 h, respectively. Hematoxylin and eosin (H&E) staining; * Hypertrophied renal glomeruli; Magnification objective 10× (**a**), 40× (**b**) and 20× (**c**).

The histopathological analysis did not record significant structural changes in the liver (Figure 2a) and heart (Figure 2b), respectively, following the administration of GdDOTA⊂CS-TPP/HA nanohydrogels after 24 and 48 h, compared to control.

In the case of the kidneys, significant histological changes were recorded in the renal cortex at 24 h after the administration of GdDOTA⊂CS-TPP/HA nanohydrogels, compared to control (Figure 2c). A hypertrophy of the renal glomeruli with vacuolation of the mesangial cells was observed. Also, the renal tubules appeared dilated, with atrophied epithelial cells, many of them having rarefied cytoplasm and apoptotic nuclei.

At 48 h after the administration of GdDOTA⊂CS-TPP/HA, the histological aspect of the kidneys was similar to the control.

In the present study, the levels of serum biochemical markers specific to liver, heart, and kidney function were kept at the control level, except for TGO and TGP which registered significant decreases at 48 h post-treatment (Table 2).

### 3.3. The Specific Superoxide Dismutase Activity (SOD), Catalase (CAT), and Glutathione Peroxidase (GPx) in Liver, Kidney, and Heart Tissues

**Figure 3:**
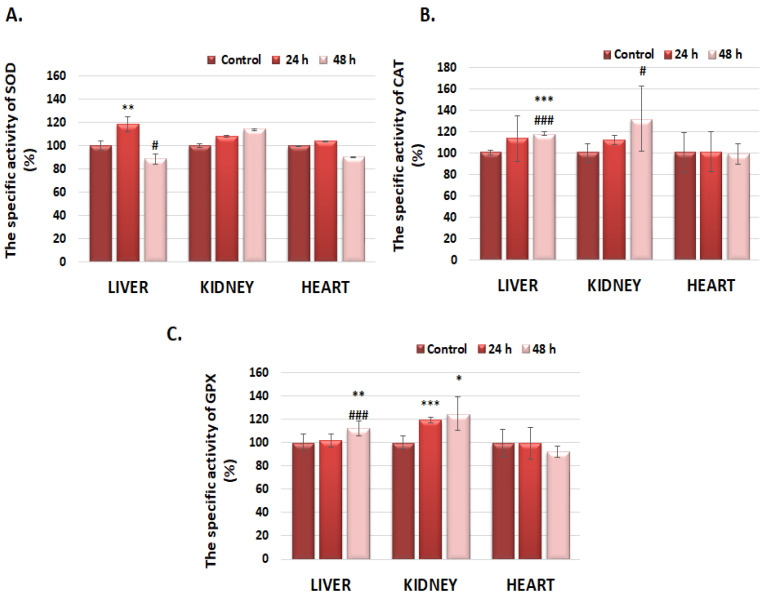
(**A**) The specific activity of superoxide dismutase (SOD), (**B**) catalase (CAT), and (**C**) glutathione peroxidase (GPx) in the liver, kidney, and heart 24 and 48 h after the subcutaneous injection of GdDOTA⊂CS-TPP/HA nanohydrogels. Data are calculated as mean value ± SD (n = 6) and expressed relative to the corresponding control. * *p* < 0.05 (significant, compared to control); ** *p* < 0.01 (distinctly significant, compared to control); *** *p* < 0.001 (very significant, compared to control); # *p* < 0.05 (significant, between the two time intervals); and ### *p* < 0.001 (highly significant, between the two time intervals).

Superoxide dismutase (SOD) specific activity in the liver increased by 19% and decreased by 12%, compared to control, after 24 and 48 h of exposure to GdDOTA⊂CS-TPP/HA, respectively (Figure 3A). Although the specific activity of SOD at the kidney level did not show changes with statistical significance, it displayed an increasing trend, being higher by 14% compared to control after the 48 h interval, a profile that could be correlated with those obtained for the antioxidant enzymes CAT and GPx. At the cardiac level, a slight decrease (by 10%) in the SOD-specific activity was also observed, similar to GPx, after the 48 h interval (Figure 3C), which could be correlated with the decrease (by 18%) in the concentration of reduced glutathione (GSH) registered at the level of the same organ (Table 3).

At 24 h after injection of GdDOTA⊂CS-TPP/HA nanohydrogels, in the liver tissue, the specific activity of catalase (CAT) registered a slight increase, by 14% compared to the control, a value that remained relatively constant 48 h after exposure (18% compared to control) (Figure 3B). A progressive increase in the specific catalase activity by 12% and 32% was noticed at the renal level, after 24 and 48 h, respectively, after injection of GdDOTA⊂CS-TPP/HA, an effect that could suggest the formation of reactive oxygen species, probably due to renal elimination of the nanogels. In the case of the heart, catalase specific activity remained constant at the control level throughout the entire treatment period (48 h).

Although in the first 24 h after injection the specific activity of GPx, at the liver level, remained unchanged compared to the control, it registered a significant increase by 12% after the interval of 48 h, reaching a level comparable to that of catalase (Figure 3C). Also, marked increases of 19% and 25% in the specific activity of GPx were highlighted at the renal level, after 24 and 48 h, respectively, data that outline an approximately similar profile with that resulting in the case of specific catalase activity.

At the cardiac level, only a slight decrease (8%) in the specific activity of GPx compared to the control was observed after the 48 h interval.

### 3.4. Evaluation of Oxidative Stress Markers

As can be seen in Table 3, the concentration of reduced glutathione (GSH) showed variations over time both in the liver and kidney, as well as in the heart. Thus, at the liver level, after injection, a progressive decrease in the concentration of GSH by 11% and 21% compared to the control, after 24 and 48 h, respectively, was noticed, while, in the kidney, a significant decrease by 30% compared to the control was highlighted after the 48 h interval.

The evaluation of the degree of lipid peroxidation induced with the GdDOTA⊂CS-TPP/HA nanohydrogels exposure in different tissues is shown in Table 3. Slight increases compared to the control level of MDA, of 9% and 11%, were noticed at the renal and, respectively, hepatic and cardiac levels, after the 48 h interval. The greater increase in the level of MDA (17%) compared to control, recorded at the cardiac level 24 h after the injection of GdDOTA⊂CS-TPP/HA, was not statistically significant. According to these data, these nanohydrogels did not induce lipid peroxidation in vivo, thus suggesting their biocompatibility.

In addition, the levels of AOPPs, in the case of the liver, kidney, and heart, remained at the control level after 24 and 48 h (Table 3). Another marker of oxidative stress is represented by protein carbonyls, compounds that can result either through the oxidative cleavage of a polypeptide chain or the direct oxidation of amino acids such as proline, arginine, lysine, and threonine [32]. The levels of carbonyl groups produced after injection of nanohydrogels in the liver and heart tissues of mice, respectively, did not show significant changes compared to control after 24 and 48 h (Table 3). At the renal level, an increase compared to control of 12% after 24 h, reaching a maximum of 19% after 48 h from injection, occurred.

### 3.5. The Analysis of Nitrosylated Protein Expression in the Liver, Kidney, and Heart via Western Blot

**Figure 4:**
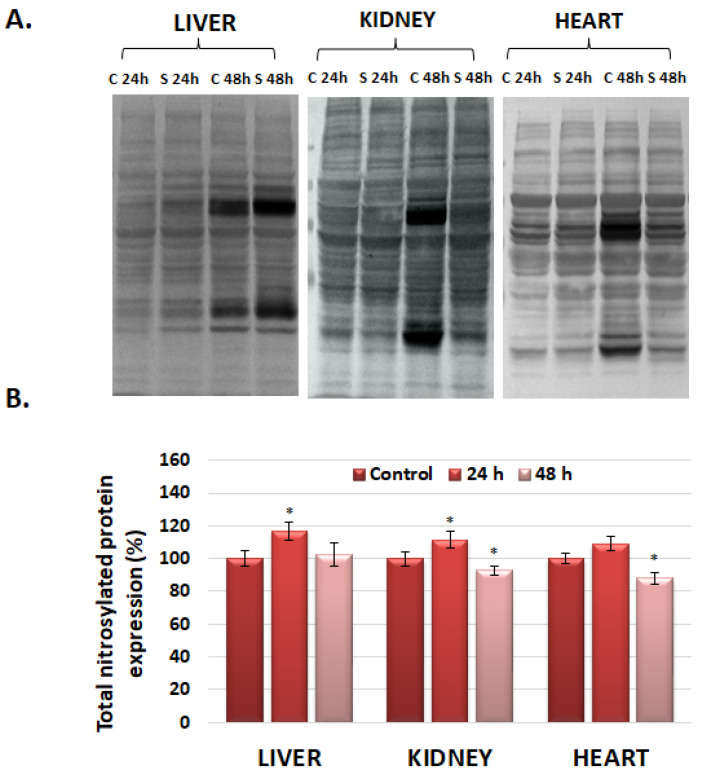
Total nitrosylated protein expression 24 and 48 h after GdDOTA NGs injection in liver, kidney, and heart. (**A**) represents the profile of nitrosylated proteins, and (**B**) represents the quantifying of the bands and the graphic representation of the protein expression compared to the control. The asterisks represent the statistical significance obtained with the Student’s *t*-test, as follows: *p* < 0.05 *; The vertical bars represent the standard deviation.

Nitric oxide plays a defining role in the regulation of redox signaling and cell function. It can be generated via NO synthases or by the breakdown of nitrite and other NO-generating compounds. S-nitrosylation is a post-translational modification characteristic of proteins that is generated due to the interaction between thiol (SH) groups of cysteine residues and nitric oxide (NO), generating S-nitrosothiol compounds [33]. The quantity of nitrosylated proteins showed an increase in the case of all analyzed organs compared to the control 24 h after the injection of nanohydrogels (Figure 4), followed by a significant decrease after 48 h in the case of the liver, kidney, and heart.

## 4. Discussion

Nanogels are drug delivery systems with improved adaptability that can absorb large amounts of water. They deliver the therapeutic agent with lower adverse effects, enabling lower therapeutic doses. They can reach small capillary vessels and penetrate the tissue either through paracellular or transcellular pathways [34]. Apart from drug delivery systems, nanogels could be used in bioimaging and biosensing [35].

The development of every pharmaceutical product requires a stage of investigative toxicology for the prediction of its clinical safety. Current strategies are represented by in silico, biochemical, and cellular in vitro assays and in vivo experiments before the experimental clinical trials [36]. The evaluation of the toxicity using in vitro models is mandatory but insufficient for anticipating possible adverse reactions in humans, with in vivo studies being necessary to elucidate the mechanisms underlying them in a complex multicellular organism [37]. In this context, the in vitro studies on the A-20, SVEC4-10, and RAW 264.7 cell lines previously published by our group [19,20,38] proved low toxicity and good biocompatibility of GdDOTA and GdDOTP NGs, and an in vivo study was mandatory.

In vivo studies have demonstrated that intravenously administered GBCAs can determine, after an interval of 48 h from injection, both biochemical and histopathological changes [39,40].

In our experiment, the serum biochemical markers that reflect the hepatic (GGT, ALP), cardiac (LDH, CK-MB), and renal function (CREA), respectively, presented no significant differences up to 48 h post exposure to GdDOTA⸦CS-TPP/HA compared to control animals. A significant decrease in TGO and TGP serum activities was noticed only after 48 h of exposure of animals to nanogels compared to the control. Probably, this is due to the interaction between the glucosamine units of chitosan, a component of the nanogels, and the aldehyde groups of pyridoxal-5’-phosphate, the coenzyme of TGO and TGP [41]. As a consequence of the reduced quantity of coenzyme, the catalytic activities of both enzymes decreased. Probably, after longer periods postexposure, due to the excretion of the NGs, these enzymatic activities could reach the same level as the control.

Since DOTA is a macrocyclic chelator, the probability of chelating Gd^3+^ ions is high [42]. Previous studies have revealed that macrocyclic GBCAs washout rapidly from rat organs and after 14 days postdosing are not detected. However, after a single human equivalent dose of GBCA in rats, Gd^3+^ was detected 20 weeks post-treatment in the kidney and liver [43].

Recently, it was reported that Gd^3+^ is a lanthanide ion that could be considered a calcium mimetic in calcium-signaling/buffering proteins [44]. Considering that Gd^3+^ inhibits Ca-dependent enzymes and voltage-activated channels, several physiological consequences could occur. Ca^2+^ is an important secondary messenger implicated in many signaling pathways and is stored mainly in the endoplasmic/sarcoplasmic reticulum (ER/SR) and Golgi apparatus, whereas mitochondria represent the Ca^2+^ buffering organelle [45].

In the cardiac tissue, inhibition of the voltage-activated channels is correlated with that of the ryanodine receptors [46] and inositol 1,4,5 triphosphate receptors. As a result, the depletion of cytosolic Ca^2+^ favors the movement of mitochondria to the ER and promotes ER–mitochondrial contacts and Ca^2+^ uptake [45]. At the same time, SERCA proteins from the ER could possibly be inhibited and depletion of this secondary messenger could occur, and, as a result, the level of mitochondrial Ca^2+^ might be diminished.

Ca^2+^ is involved in the activation of α-ketoglutarate dehydrogenase and isocitrate dehydrogenase in the Krebs cycle, which generates NADH, and oxidative phosphorylation in the mitochondria. Low levels of Ca^2+^ could diminish NADH generation and a decrease in superoxide production at the level of complexes I and III of the mitochondrial electron transporter chain [47]. As a result, the level of superoxide, a substrate of SOD enzymes, could decrease, and this was probably the reason why the total SOD activity diminished in the heart of mice 48 h after exposure to GdDOTA⸦CS-TPP/HA compared to control animals. Probably this decrease is insignificant because mitochondria represent one third of cardiac cell volume, reflecting the high energy demand in the heart [48]. The reaction product, hydrogen peroxide, was decomposed in the reactions catalyzed by CAT and GPx. These activities were not different from controls. In liver cells, Ca^2+^ permeable channels in the plasma membrane are critical for hepatocytes’ physiology and are of different types: store-operated, ligand-gated, and receptor-activated, as well as stretch-activated [49]. The alteration of the function of these channels could decrease the cytosolic concentration of Ca^2+^, and, as a consequence, different isoforms of phospholipase C could be inhibited and lower IP3 concentration would be produced. As a result, the release of Ca^2+^ from the ER could slow, and, together with possible inhibition of SERCA isoforms, a lower concentration in the intermembrane space of mitochondria could be generated that could affect the conformation of proteins belonging to complexes I and III, and, probably, a higher quantity of superoxide was formed. A higher substrate quantity was correlated with an increase in total SOD activity, which catalyzes the reaction of dismutation of superoxide in hydrogen peroxide. This was decomposed in the reactions catalyzed by CAT and GPx. After 24 post-treatment, CAT activity increased insignificantly but after 48 h, both CAT and GPx activities increased significantly to decompose the oxidant hydrogen peroxide.

The kidney presents a unique vascular structure, and, post administration of GBCAs, an increase in the blood viscosity could occur, with an impact on blood flow dynamics that could produce ischemic damage to the renal tubules [50]. In the case of ischemia, the reduction in oxygen supply generates the switch from aerobic to anaerobic metabolism and mitochondrial dysfunction with overproduction of ROS [51]. Taking into account that the total SOD activity was slightly increased up to 48 h post-treatment, probably no significant ischemic process occurred, and an insignificant quantity of hydrogen peroxide was produced. But the kidney is one of the richest organs in peroxisomes, organelles that contain a lot of enzymes involved in fatty acid oxidation, amino acid metabolism, and plasmalogen synthesis. Hydrogen peroxide is an abundant product of some of these metabolisms [52]. Probably in the presence of NGs, the generation of these compounds and consequently CAT and GPx activities increased in a time-dependent manner to diminish the quantity of this antioxidant.

Superoxide is very reactive and attacks the methylene group between the double bonds of polyunsaturated fatty acids, initiating lipid peroxidation. The marker of this process is the generation of MDA. According to our data, the MDA level did not change significantly after 24 h and 48 h of administration, suggesting that the enzymatic antioxidant system succeeded in counteracting ROS.

ROS can also affect proteins. AOPP and PCG are well-known markers of the oxidative modifications of proteins. AOPPs are formed in the presence of hypochlorous acid with proteins and they are represented especially by dityrosine. PCG results due to the direct oxidation of lysine, arginine, proline, and threonine residues or by the generation of adducts with MDA and other lipid oxidation products [53]. In our experiment, no variation of the concentrations of AOPP and PCG compared to control was noticed after injection of GdDOTA⸦CS-TPP/HA.

Apart from enzymatic antioxidants, nonenzymatic ones also play an important role in the defense against ROS. GSH is the most important nonenzymatic antioxidant. It is a substrate for GPX and glutathione-S-transferase, but it can also interact nonenzymatically with heavy metals and other chemical compounds. Our study showed that the GSH level diminished significantly 48 h after exposure to GdDOTA⸦CS-TPP/HA compared to control. This can be correlated with the increase in GPX activity in the liver and kidney. In the heart, the decrease in GSH concentration was the lowest compared with the other two analyzed organs, suggesting that this organ is protected to a certain extent against the oxidative stress induced by GdDOTA⸦CS-TPP/HA systemic administration.

Previous studies revealed that exposure of macrophages to GBCAs generated NO and proinflammatory cytokine production [54]. NO is synthesized starting from arginine in the reaction catalyzed by NO synthases (NOS). There are three types of enzymes: neuronal NOS (nNOS or NOS1), inducible NOS (iNOS or NOS2), and endothelial NOS (eNOS or NOS3). In physiological conditions, the constitutive isoforms nNOS and eNOS produce low amounts of NO. However, under cytokine stimulation, iNOS is activated and generates high levels of NO. Superoxide can react with NO producing a peroxynitrite anion (ONOO^−^) that can nitrate several biomolecules, including proteins, to produce 3-nitrotyrosine, a biomarker of the nitrosative stress. This post-translational modification can promote the inactivation of functional proteins and enzymes and can generate pathological conditions in all organs, such as alterations in mitochondrial energy metabolism, transcription, and ion channel functions [55].

Our data revealed that the nitrosative stress appeared 24 h after exposure in all three organs but it was counteracted by the enzymatic and nonenzymatic antioxidative system, with the level of nitro-tyrosine being decreased under control levels 48 h after exposure.

A limited number of in vivo studies have addressed the effects of gadolinium-based nanoparticles on redox status at the tissue level [56]. To explore the antioxidant capacity of [Gd@C_82_(OH)_22_]n nanoparticles, Wang et al. evaluated, among others, the activities of the antioxidant enzymes SOD, CAT, GPx, and glutathione S transferase (GST), as well as the levels of GSH, MDA, and protein thiols, from liver tissue samples, from tumor-bearing mice (induced via subcutaneous implantation of murine hepatoma cells, H22), and from normal mice (control group) [56]. According to the obtained data, the significant increases in enzyme activities and other oxidative stress parameters, recorded following the inoculation of H22 cells in normal mice, were reduced after treatment with [Gd@C_82_(OH)_22_]n, in a dose of 31.4 μg Gd/kg (2 × 10^−7^ mol/kg), with a tendency to return to normal levels, with the nanoparticles demonstrating the ability to regulate ROS production in vivo.

Returning to our data, the efficiency of counteracting oxidative stress could be due to the antioxidant activity of the components of the polymeric matrix of NGs, i.e., chitosan [57] and hyaluronic acid [58].

The potential of GdDOTA⸦CS-TPP/HA nanohydrogels to cause alterations, inflammation, or damage in mouse tissues (kidney, liver, and heart) in vivo was investigated with histological analysis. The significant structural changes highlighted at the renal level 24 h after the administration of GdDOTA⊂CS-TPP/HA, compared to the control, could be supported by the supposition that the generated ROS and NO could affect the kidney tissue architecture. But the increase in SOD, CAT, and GPX activities alleviated these alterations later. Also, probably the renal elimination of these NGs occurred after 48 h and the histological aspect of the kidney became like the control ones. In addition, the lack of histological changes in the liver and cardiac tissues, respectively, as well as in the lungs (Figure S1, Table S1), could suggest the capacity of these organs to counteract the toxicity of nanohydrogels. These histopathological analysis data are consistent with those obtained with other MRI contrast agents [59,60,61,62].

## 5. Conclusions

The present work includes a preliminary in vivo study regarding the biocompatibility of gadolinium-based nanohydrogels, designed as contrast agents for MRI applications, in which CD-1 albino mice served as animal models. According to our data, the formulation of GdDOTA⊂CS-TPP/HA NGs presented good biocompatibility at the level of mice liver, kidney, and heart and could be a good candidate for a new MRI contrast agent with low toxicity.

## Figures and Tables

**Table 1:** Physico–chemical characteristics of GdDOTA⊂CS-TPP/HA nanogels (NGs) according to chitosan (CS) concentration in the citric acid phase.

[CS] mg/mL	2.5
D_H_ (nm)	217
PdI	0.2
ζ (mV)	30.3
[Gd]_t_ M	9.87 × 10^−5^

Note: [CS]—chitosan concentration; D_H_—hydrodynamic diameters; PdI—polydispersity index; ζ—Zeta Potential; [Gd]_t_—total concentration of gadolinium.

**Table 2:** Biochemical blood markers after 24 and 48 h from exposure of GdDOTA nanohydrogels.

Markers	24 h		48 h	
	Control Group	Exposed Group	Control Group	Exposed Group
TGO (U/L)	88.58 ± 14.36	83.73 ± 15.86	83.43 ± 27.28	53.31 ± 19.29
TGP (U/L)	26.25 ± 9.61	25.71 ± 9.85	28.6 ± 5.36	18.21 ± 3.45
LDH (U/L)	2229.95 ± 71.08	2252.96 ± 70.35	885.73 ± 43.35	681.53 ± 181.99
GGT (U/L)	2.46 ± 0.23	2.56 ± 0.18	3.01 ± 0.30	2.75 ± 0.30
ALP (U/L)	96.26 ± 33.56	105.86 ± 51.40	104.56 ± 49.25	110.18 ± 40.44
CREA (mg/dL)	0.29 ± 0.05	0.30 ± 0.02	0.24 ± 0.02	0.26 ± 0.02
CK-MB (U/L)	217.50 ± 38.65	222.42 ± 39.18	89.79 ± 22.20	100.73 ± 31.87

**Table 3:** The relative values of malondialdehyde, reduced glutathione, advanced oxidation protein products (AOPP), and protein-reactive carbonyl groups (PRCG) in the liver, kidney, and heart of CD-1 albino mice after subcutaneous injection of GdDOTA⊂CS-TPP/HA nanohydrogels.

Oxidative Stress Markers	Organ	24 h		48 h	
		Control Group	Exposed Group	Control Group	Exposed Group
MDA (nmoles/mg)	Liver	100 ± 12.06	103.23 ± 10.22	100 ± 18.06	111.32 ± 10.33
	Kidney	100 ± 6.73	108.60 ± 9.37	100 ± 13.81	109.04 ± 20.00
	Heart	100 ± 11.10	117.24 ± 17.78	100 ± 2.98	116.96 ± 13.92
GSH (nmoles/mg)	Liver	100 ± 26.40	88.93 ± 15.90	100 ± 47.29	79 ± 16.67
	Kidney	100 ± 26.58	94.97 ± 19.67	100 ± 17.13	69.60 ± 19.00
	Heart	100 ± 18.44	99.71 ± 11.88	100 ± 14.80	82.46 ± 6.94
AOPP (µmoles/mg)	Liver	100 ± 13.17	104.53 ± 11.39	100 ± 13.17	105.24 ± 16.25
	Kidney	100 ± 3.30	98.44 ± 19.36	100 ± 7.33	97.65 ± 7.33
	Heart	100 ± 3.77	97.73 ± 5.98	100 ± 11.75	100.46 ± 7.33
PCG (nmoles/mg)	Liver	100 ± 10.47	99.76 ± 7.65	100 ± 9.17	98.35 ± 12.59
	Kidney	100 ± 9.76	112.56 ± 13.43	100 ± 9.44	118.95 ± 15.91
	Heart	100 ± 14.75	98.76 ± 7.35	100 ± 2.97	98.47 ± 2.97

## Data Availability

Data are contained within the article and supplementary materials.

## Supplementary Materials

The following supporting information can be downloaded at: https://www.mdpi.com/article/10.3390/polym16081064/s1, Figure S1: The specific activity of superoxide dismutase (SOD), catalase (CAT) and glutathione peroxidase (GPx) in the lung after 24 and 48 h after the subcutaneous injection of GdDOTA⊂CS-TPP/HA nanohydrogels. Data are calculated as mean value ± SD (n = 6) and expressed relative to the corresponding control; Table S1: The relative values of malondialdehyde, reduced glutathione, advanced oxidation protein products (AOPP) and protein reactive carbonyl groups (PRCG) in the liver, kidney and heart of CD-1 albino mice after subcutaneous injection of GdDOTA⊂CS-TPP/HA nanohydrogels.
