# Supplementary material for: In Vivo Evaluation of Innovative Gadolinium-Based Contrast Agents Designed for Bioimaging Applications
Source: Polymers (Basel). 2024 Apr 11;16(8):1064. doi: 10.3390/polym16081064 (PMC11054998; doi:10.3390/polym16081064)
Supplement: Supplementary file 1 [file polymers-16-01064-s001.zip › polymers-2918476-supplementary.pdf]

Supplementary material

In vivo evaluation of innovative Gadolinium Based Contrast Agents designed for Bioimaging Applications

Sorina Nicoleta Voicu, Cecilia Virginia Gheran, Cornel Balta, Anca Hermenean, Maité Callewaert, Françoise Chuburu and Anca Dinischiotu

Table of contents:

- Figure S1
- Table S1

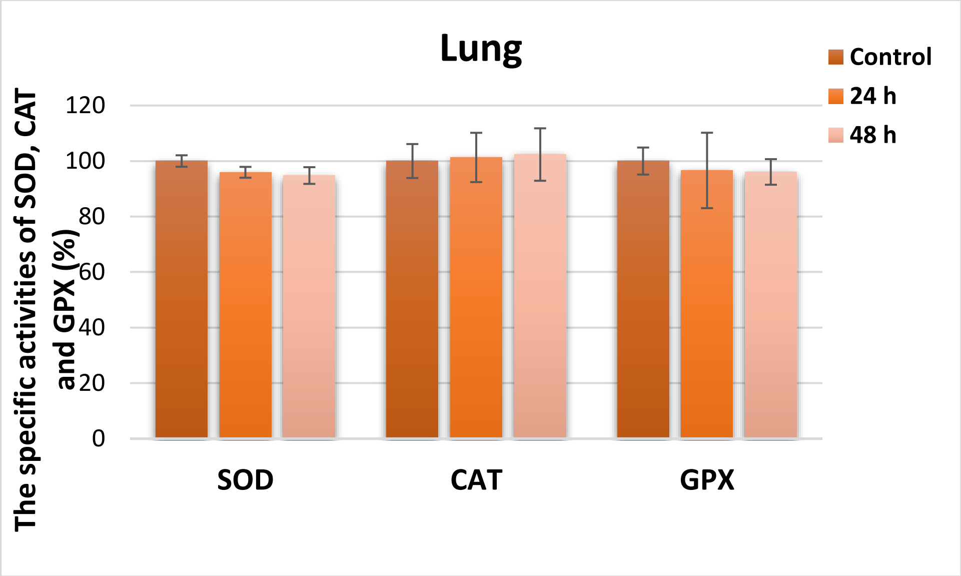

Figure S1. The specific activity of superoxide dismutase (SOD), catalase (CAT) and glutathione peroxidase (GPx) in the lung after 24 and 48 hours after the subcutaneous injection of GdDOTA⊂CS-TPP/HA nanohydrogels. Data are calculated as mean value ± SD (n=6) and expressed relative to the corresponding control.

Table 1. The relative values of malondialdehyde, reduced glutathione, advanced oxidation protein products (AOPP) and protein reactive carbonyl groups (PRCG) in the liver, kidney and heart of CD- 1 albino mice after subcutaneous injection of GdDOTA⊂CS-TPP/HA nanohydrogels.

**Oxidative stress markers**	**Organ**	**24 h**		**48 h**	
		**Control group**	**Exposed group**	**Control group**	**Exposed group**
**MDA (nmoles/mg)**	**Lung**	100 ± 12.3	100.56 ± 2.3	100 ± 6.3	111.51± 7.25
**GSH (nmoles/mg)**	**Lung**	100 ± 8.9	106.4 ± 4.5	100 ± 7.1	107.93 ± 6.67
**AOPP (µmoles/mg)**	**Lung**	100 ± 7.8	109.49 ± 10.39	100 ± 14.2	111.10 ± 6.25
**PCG (nmoles/mg)**	**Lung**	100 ± 6.7	103.71 ± 7.65	100 ± 9.17	106.6 ± 12.59
